# The effect of aqueous extract of *Arctium lappa* root on the survival of *Lactobacillus acidophilus* La‐5 and *Bifidobacterium bifidum* Bb‐12 and sensorial and physicochemical properties of synbiotic yogurt

**DOI:** 10.1002/fsn3.3919

**Published:** 2023-12-29

**Authors:** Elmira Vanaki, Abolfazl Kamkar, Negin Noori, Asghar Azizian, Fatemeh Mohammadkhan

**Affiliations:** ^1^ Department of Food Hygiene & Control, Faculty of Veterinary Medicine University of Tehran Tehran Iran

**Keywords:** antioxidant activity, *Arctium lappa* root, probiotic bacteria, survival, yogurt

## Abstract

The effect of aqueous extract of *Arctium lappa* root (ALE) on the survival of *Lactobacillus acidophilus* La‐5 and *Bifidobacterium bifidum* Bb‐12 probiotic bacteria and sensory and physicochemical properties of synbiotic yogurt was evaluated during 4 weeks storage at 4°C. According to this study, using 0.5% and 1% ALE significantly affected the survival of La‐5 and Bb‐12 during storage. The results showed that 1% of ALE counting of La‐5 and Bb‐12 has been reached from 6.96 and 8.14 Log CFU/g to 7.3 and 7.30 Log CFU/g after 28 days of storage. Moreover, adding 1% ALE to yogurt enhanced antioxidant activity and phenolic content to 1299.8 mg gallic acid/kg and 392.8 mg BHT eq./kg compared with the control (without extract) after storage, respectively. In general, in yogurt containing ALE, a decrease in Syneresis, undesirable changes in taste, texture, and appearance, and reduced overall acceptances were observed compared to the control. In conclusion, using this prebiotic compound (ALE) can improve nutritional properties and probiotic protection in yogurt during long time storage; thus, it is a good choice for application in the dairy industry.

## Graphical abstract

The present study was designed to investigate the effect of aqueous extract of *Arctium lappa* root on the survival of *Lactobacillus acidophilus* La‐5 and *Bifidobacterium bifidum* Bb‐12 and sensorial and physicochemical properties of synbiotic yogurt. According to the results of the present study, using an aqueous extract of *Arctium lappa* root can improve nutritional properties and probiotic protection in yogurt during long time storage; thus, it is a good choice for application in the dairy industry.

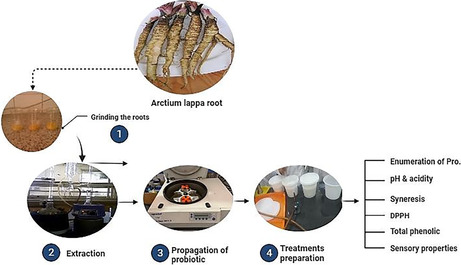

## 1 INTRODUCTION

Fortunately, various probiotic products are available around the world today. Yogurt is one of the products that has attracted a lot of attention as a potential probiotic product (Madhu et al., 2012). Probiotic yogurt not only provides beneficial bacteria for the human body but is also a source of valuable nutrients such as calcium and biological peptides. Hence, probiotic yogurts are gaining an important position in the global market of food products (Madhu et al., 2012). Several studies have reported the benefits of probiotics and their health effects (Daliri & Lee, 2015; Nagpal et al., 2012). These include: reducing the incidence of diarrhea, increasing the resistance of the immune system, lowering blood cholesterol, and improving the symptoms of lactose intolerance and antitumor effects (Noland & Aryana, 2012). Despite the lack of general consensus on the minimum number of viable cells of probiotics to produce their health‐giving effects, the number of 10^6^–10^8^ cfu/mL or cfu/g has been accepted as a satisfactory level (Mortazavian et al., 2012). *Lactobacillus acidophilus* and *Bifidobacterium bifidum* are the most common probiotic bacteria in many probiotic foods, especially yogurt. High bioavailability and irritability, creating a creamy texture, improving the flavor and texture properties, and producing short‐chain fatty acids are the benefits of using these probiotics in food, especially dairy products (Madhu et al., 2012). The potential effects of a probiotic product can be generated when the number of probiotic bacteria is not less than a certain limit. Accordingly, the survival of probiotic bacteria is important and significant. Manufacturers of probiotic products always use new methods to increase the survival of bacteria because the survival and growth rate of bacteria in the probiotic product diminish over time due to improper storage conditions (Madhu et al., 2012; Mortazavian et al., 2012).

One of these methods is using plant extracts with medicinal properties as food for probiotics. “Prebiotics” are called probiotic foods. Consumption of these compounds can lead to the growth of beneficial gastrointestinal bacteria or probiotics (Di Criscio et al., 2010; Walker & Duffy, 1998). Inulin, soybean oligosaccharides, fructooligosaccharides, and galactooligosaccharides are common prebiotics used in probiotic products (Schrezenmeir & de Vrese, 2001). Because of the association between probiotics and prebiotics, foods containing both are called “synbiotics” (Di Criscio et al., 2010; Walker & Duffy, 1998). Most herbs that contain compounds such as inulin and cellulose fibers can be used as beneficial prebiotics. *Arctium lappa* is an herbaceous plant that grows wild in some humid and temperate regions of Iran. This plant has a snake‐like stem covered with rough hairs, and its vast and wide leaves lie on it (Ahangarpour et al., 2013; Azizov et al., 2012). The root of this plant is long and spindle shaped and has healing properties. *Arctium lappa* root has brown skin, white inside, and a sweet taste but unpleasant odor. *Arctium lappa* root contains large amounts of inulin (up to 45%) and other compounds such as tannins and flavonoids (0.7%), carotenoids (13.2%), fatty oil (up to 3.9%), protein (12.3%), iron (0.027 mg/g), potassium (7.47 mg/g), magnesium (0.39 mg/g), oleic acid, linoleic acid, and vitamins C and B6. Due to the presence of these nutrients in Baba Adam root, it can be used as a food source to feed probiotic bacteria and prevent their survival (Ahangarpour et al., 2013; Azizov et al., 2012). According to our latest information, no study has been done on the antioxidant and probiotic function of *Arctium lappa* root in yogurt. Hence, this study was designed to investigate the performance of different percentages of the extract prepared from the *Arctium lappa* root on the growth and survival of two prominent and widely used probiotic bacteria in dairy products and probiotic products (La‐5 and Bb‐12) as well as some physical, chemical and organoleptic characteristics in the samples yogurt.

## 2 MATERIALS AND METHODS

### 2.1 Collection and identification of *Arctium lappa*

*Arctium lappa* root was collected from the natural resources of Isfahan province in late August 2021. The samples were identified by the botanist of the Herbarium Center of the Faculty of Pharmacy, University of Tehran, and registered with Herbarium number (L.357¬3).

### 2.2 Aqueous extract of *Arctium lappa*

Five hundred gram of dried *Arctium lappa* root was crushed by a mill (IKA M20 universal) and poured into a Zimax, then 2 L of water was added. The Erlenmeyer was placed (T&N) on the shaker for 24 h, and after 24 h, two filter papers were used to filter the contents. In the next step, the filtered extract was dried at 40°C with the help of a rotary operator (Sterogla, STRIKE 100). Finally, after a week, the dried extract was collected and stored in sterile colored glass jars at refrigerator temperature until use (Azizov et al., 2012; Faraki et al., 2020).

### 2.3 Propagation of probiotics

The first stage was the preparation of yogurt, for which the starter produced by Hansen (CHR Hansen) was purchased. Its microbial composition contained *Streptococcus salivarius* subsp. *thermophilus* and *L. delbrueckii* subsp. *bulgaricus* and pure freezing cultures of La‐5 and Bb‐12. In order to activate probiotic bacteria, MRS (Man–Rogasa–Sharpe) broth culture medium (Merck, Germany) is used at a temperature of 37°C for 24 h.

The culture was then incubated for another 48 h to obtain many active probiotics. Afterward, the probiotic biomass in the late‐log phase was separated by centrifugation (AG Eppendorf) at 4000*g* for 10 min at 4°C, and then washed with 9% sterile saline under the same centrifugation conditions (washing was done twice) (Homayouni et al., 2008; Noori et al., 2017). In the next step, to produce a probiotic suspension, 2 mL of sterile physiological saline solution was added to the tubes (Noori et al., 2017). Finally, the inoculation was adjusted to the required 10^9^ CFU/mL concentration using a spectrophotometer (BECKMAN).

### 2.4 Yogurt and treatments preparation

After heating 4 liters of pasteurized and homogenized milk (3.5% fat and 3.5% protein), 60 g of powdered milk was added to increase the nonfat dry matter content to 1.5%. When the milk boils for 5 min at 95–90°C, it rapidly cools to 42°C. At this temperature, yogurt starter bacteria (2% w/v) belonging to the Italian Dalton Biotecnologie Company, 10^9^ CFU/mL of probiotic bacteria, and then 0.5% and 1% aqueous extract of ALE were added (Table 1). The mixture was stirred, distributed in 100 mL sterile jars, and incubated at 42°C until pH reached 4.5–4.6. After incubation, yogurt samples were stored at 4°C until the tests. All experiments were repeated twice (Bertrand‐Harb et al., 2003; Faraki et al., 2020).

**TABLE 1:** Different treatments in this study.

Row	Treatments	Description
1	Control	Yogurt
2	Probiotic	Yogurt + *Lactobacillus acidophilus* La‐5 and *Bifidobacterium bifidum* Bb‐12
3	Probiotic + 0/5% extract	Yogurt + *Lactobacillus acidophilus* La‐5 and *Bifidobacterium bifidum* Bb‐12 + 0/5% extract
4	Probiotic + 1% extract	Yogurt + *Lactobacillus acidophilus* La‐5 and *Bifidobacterium bifidum* Bb‐12 + 1% extract

### 2.5 Enumeration of probiotic bacteria

Probiotic bacteria were counted according to standard counting techniques. For this purpose, 10 g of each sample was mixed with 90 mL of sterile peptone water until uniform (0.1% w/v). After preparing other serial dilutions, the amount of 0.1 mL of the selected dilution of *L. acidophilus* and *B. bifidum* was added to plates containing MRS (Merck) which were, respectively, prepared with bile agar for *L. acidophilus* and 0.05% L‐cysteine and 0.3% propionate sodium transferred. Then, the bacteria were incubated aerobically (La‐5) and anaerobically (Bb‐12) using a gas pack (Merck) and anaerobic glass at 37°C for 72 h. After the end of incubation, those plates with 25–250 colonies were selected for counting and finally, the results were reported in terms of cfu/g (Faraki et al., 2020; Vinderola & Reinheimer, 1999).

### 2.6 pH and titrable acidity

A digital pH meter was used to measure the pH of yogurt samples (Jenway). Titratable acidity was measured by titrating (10 g of each sample was mixed with 90 mL of sterile distilled water until uniform) using normal sodium hydroxide solution (0.1 N) and phenolphthalein indicator, then the results were reported in terms of lactic acid per liter of food (Zainoldin & Baba, 2009). Titratable acidity was calculated using the following equation:

$$\text{Titratable acidity}=\left(V\times 0.009\times 100\right)/m$$

*V* = NaOH
*m* = Sample weight (g).

### 2.7 Syneresis

To measure the syneresis test, 20 g of yogurt was taken from the samples and centrifuged in a refrigerated centrifuge at 1792 g for 15 min, and then the process of separation of the supernatant liquid was completely carried out and weighed (Sahan et al., 2008). Finally, the percentage of syneresis was calculated according to the following formula:

$$\text{Syneresis}=\left(W_{t}/W_{i}\right)$$

*Wt*: Pellet weight (g)
*W*_*i*_: Sample weight (g)

### 2.8 DPPH radical scavenging activity

For this purpose, yogurt samples were diluted, and 0.1 of it were taken and mixed with 4 mL of DPPH solution (2,2‐diphenyl‐1‐picrylhydrazil) 0.004% (w/v). Also, solutions of synthetic antioxidant butylated hydroxy toluene (BHT) in methanol solvent with different concentrations (2000, 1500, 1000, 500, 250, 125, 62, and 31 ppm) were prepared. Then, 0.1 mL of different concentrations were taken, 4 mL of DPPH solution was added, and mixed well. After 30 min, the absorbance was read at 517 nm in the spectrophotometer. DPPH radical scavenging activity was calculated from the following formula:

$$\text{DPPH radical scavenging activity}\;\left(\%\right)=\left(A_{\text{blank}}-A_{\text{sample}}/A_{\text{blank}}\right)\times 100$$

*A*_blank_ is the absorbance of the control (containing all reagents except the sample), and *an* example is the absorbance of the model (Brand‐Williams et al., 1995; Burits & Bucar, 2000; Faraki et al., 2020).

### 2.9 Total phenolic content

The phenolic compounds were measured by Folin–Ciocalteu method. For yogurt samples, the first 5 g of yogurt was diluted with 15 mL of distilled water and centrifuged for 10 min (4000 rpm), then 0.1 mL of the supernatant was removed and poured into separate tubes, and sodium bicarbonate 2% and 4 mL of Folin–Ciocalteu reagent were added. Afterward, the samples were incubated in a dark place at 25°C for 2 h, and absorbance was read by spectrophotometry at 750 nm (BECKMAN). Total phenols were expressed as mg gallic acid/kg of yogurt (Faraki et al., 2020; Vasco et al., 2008).

### 2.10 Sensory properties

Seven trained female panelists performed a sensory evaluation of the yogurt samples and were called from the food hygiene department. The appearance, texture, taste, and overall acceptability were assessed using five hedonic scales; 5 indicated like significantly, 4 for like moderately, 3 for neither like nor dislike, 2 for dislike instead, and 1 stated oppose extremely. The sensory evaluation was performed on 1st day of storage (Faraki et al., 2020; Sahan et al., 2008).

### 2.11 Statistical analysis

The collected data were analyzed using SPSS version 20.20. The one‐way analysis of variance (ANOVA) was used to determine the statistically significant difference among treatments for microbial and chemical analysis. Duncan multiple‐comparison test (MCT) was used to compare means when the difference among treatments was substantial. Kruskal–Wallis nonparametric test analyzed the sensory evaluation. The difference among treatments was considered statistically significant when *p* < .05. The final results were expressed as mean ± standard deviation (Mean ± SD).

## 3 RESULTS

### 3.1 Survival of probiotics

**FIGURE 1:**
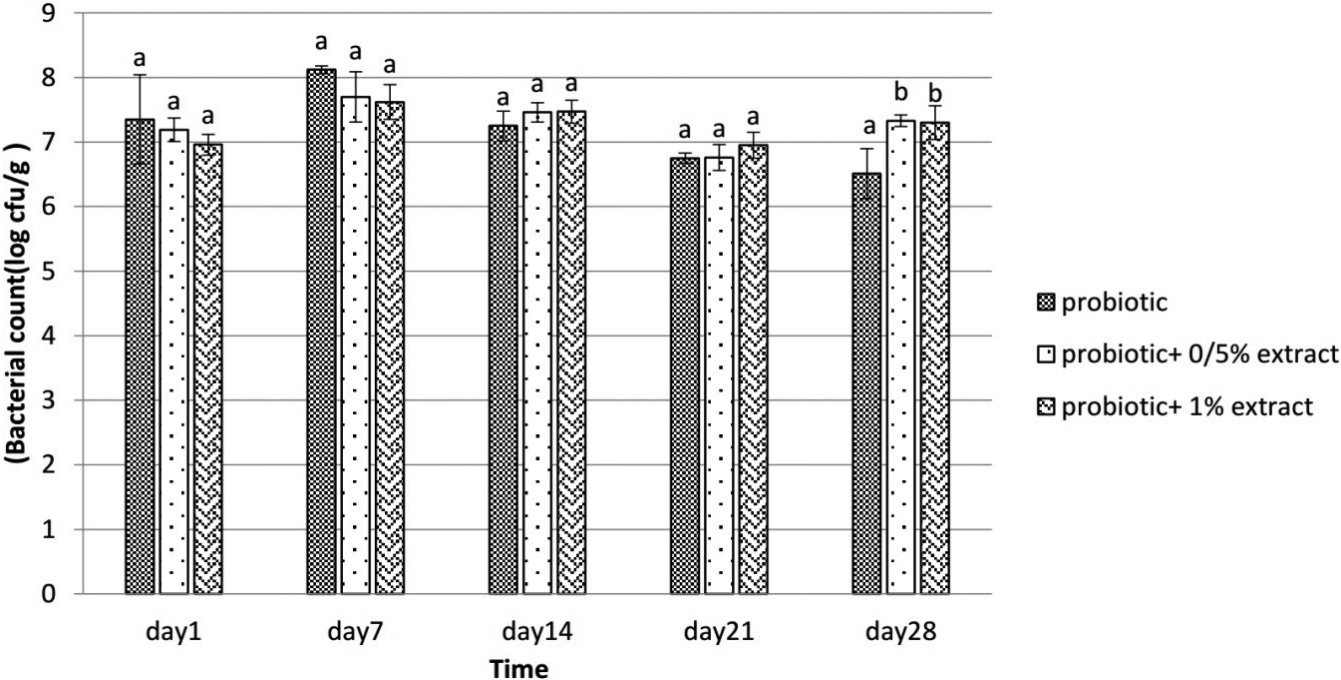
The effect of aqueous extract of *Arctium lappa* root (ALE) on the viability of *Lactobacillus acidophilus* in studied groups during 28 days of storage at 4°C. Values followed by different lowercase letters on the same days are significantly different.

**FIGURE 2:**
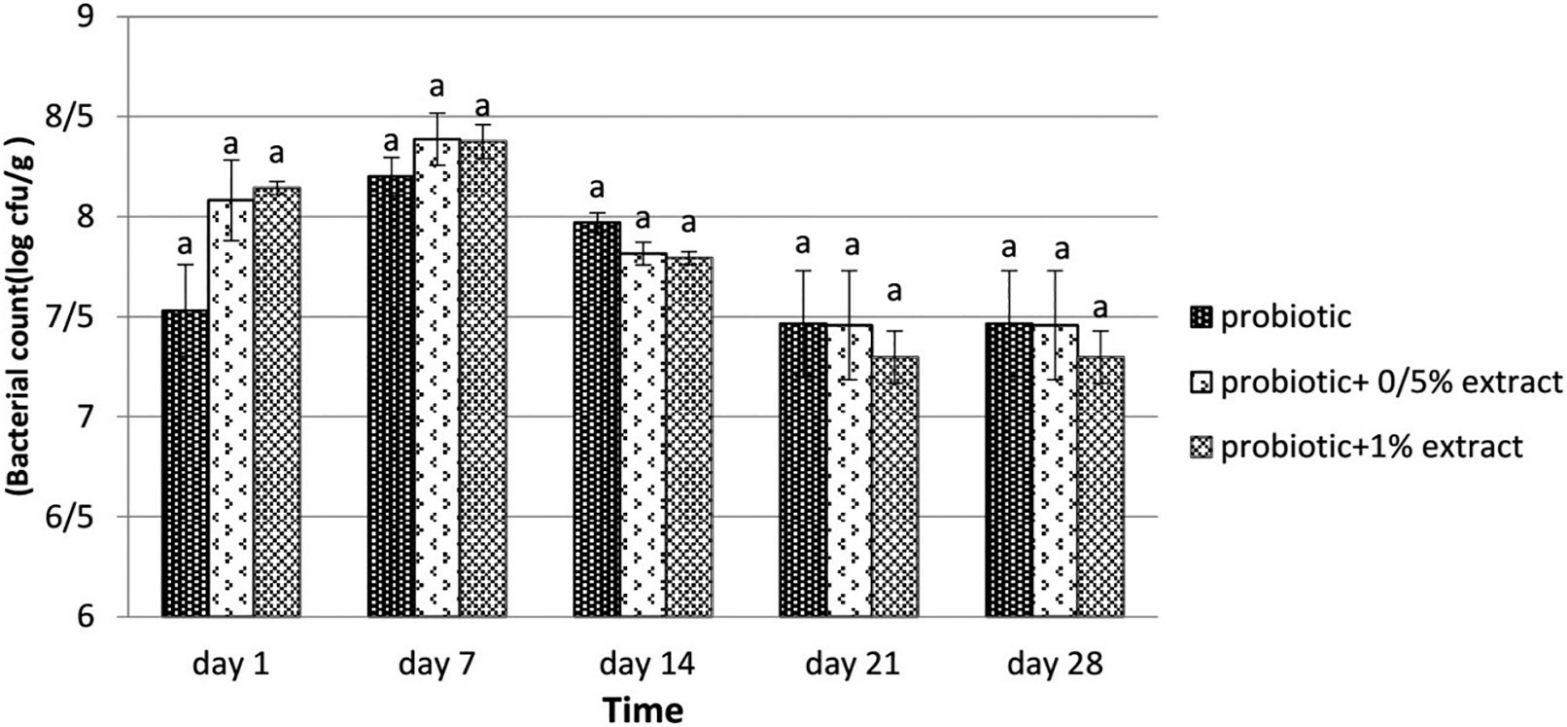
The effect of aqueous extract of *Arctium lappa* root (ALE) on the viability of *Bifidobacterium bifidum* in studied groups during 28 days of storage at 4°C. Values followed by different lowercase letters on the same days are significantly different.

Survival of La‐5 and Bb‐12 during 28 days of storage is presented in Figures 1 and 2, respectively. Based on the results, the log number of viable cells of La‐5 in probiotic yogurt containing 0.5% ALE on the first day was 7.19 cfu/g, and on the last day was 7.33 cfu/g. Also, in the group including 1% ALE, the bacterial count increased from 6.96 cfu/g on day 1 to 7.3 cfu/g on day 28. The results showed no significant difference between the group containing 0.5% and 1% ALE (*p* > .05). However, these two groups differed significantly from those containing probiotics (*p* < .05). According to the results depicted in Figure 2, there was no significant difference in Bb‐12 count in samples of yogurt treated with probiotics containing 0.5% and 1%, and probiotics without ALE for 28 days at 4°C. So in probiotic yogurt samples without extract, the count of Bb‐12 on the first day was 7.53 cfu/g, and on the 28th day was 7.47 cfu/g. In probiotic yogurt containing 0.5% of the section, the bacterial count decreased from 8.08 (log cfu/g) to 7.46, and in the group comprising 1% of the extract, from 8.14 cfu/g on the first to 7.30 cfu/g on the 28th day at 4°C.

### 3.2 The effect of aqueous extract of *Arctium lappa* root on the physicochemical properties of synbiotic yogurt

#### 3.2.1 Total phenolic content

**FIGURE 3:**
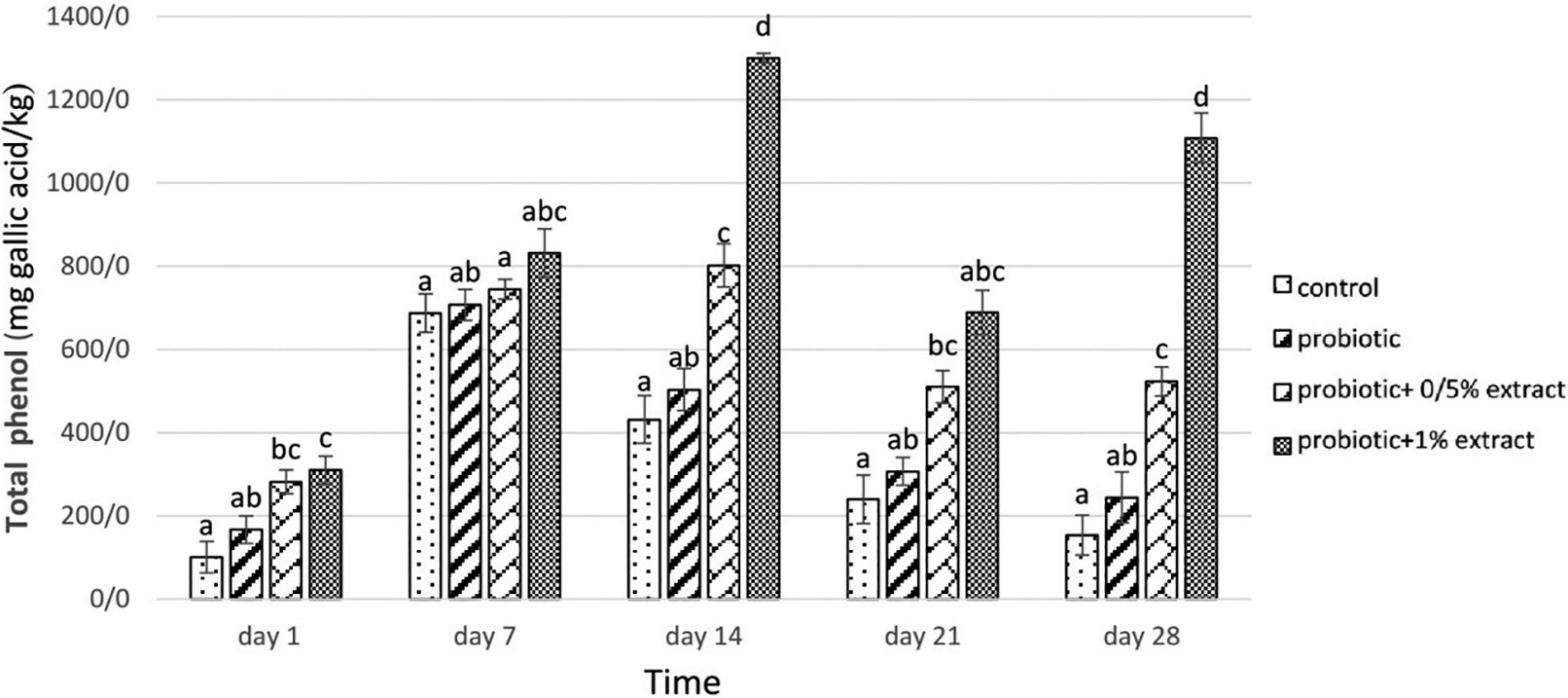
The effect of aqueous extract of *Arctium lappa* root (ALE) on the total phenolic content in studied groups during 28 days of storage at 4°C. Values followed by different lowercase letters on the same days are significantly different.

First, it is worth mentioning that the phenolic content is 0.312 mg gallic acid/mL. The results of the total phenolic content of different groups are shown in Figure 3. In this test, no significant difference in entire phenol content was observed between the control group (without probiotics and extracts) and the probiotic yogurts (*p* > .05). Adding 0.5% ALE increased the total phenol in probiotic yogurt, ranging between 281.7 and 801.6 mg gallic acid/kg during the storage time. With increasing the concentration of the ALE to 1%, a significant increase in the total phenol was observed (increased to 1299.8 mg gallic acid/kg on day 28). In general, there was a substantial difference in the entire phenol content in different treatments of yogurt treated with probiotics containing 0.5% and 1% of ALE during the study period. In addition, there was a strong positive correlation between phenolic content and ALE concentration (*r* = .904, *p* < .05).

#### 3.2.2 DPPH radical scavenging activity

**FIGURE 4:**
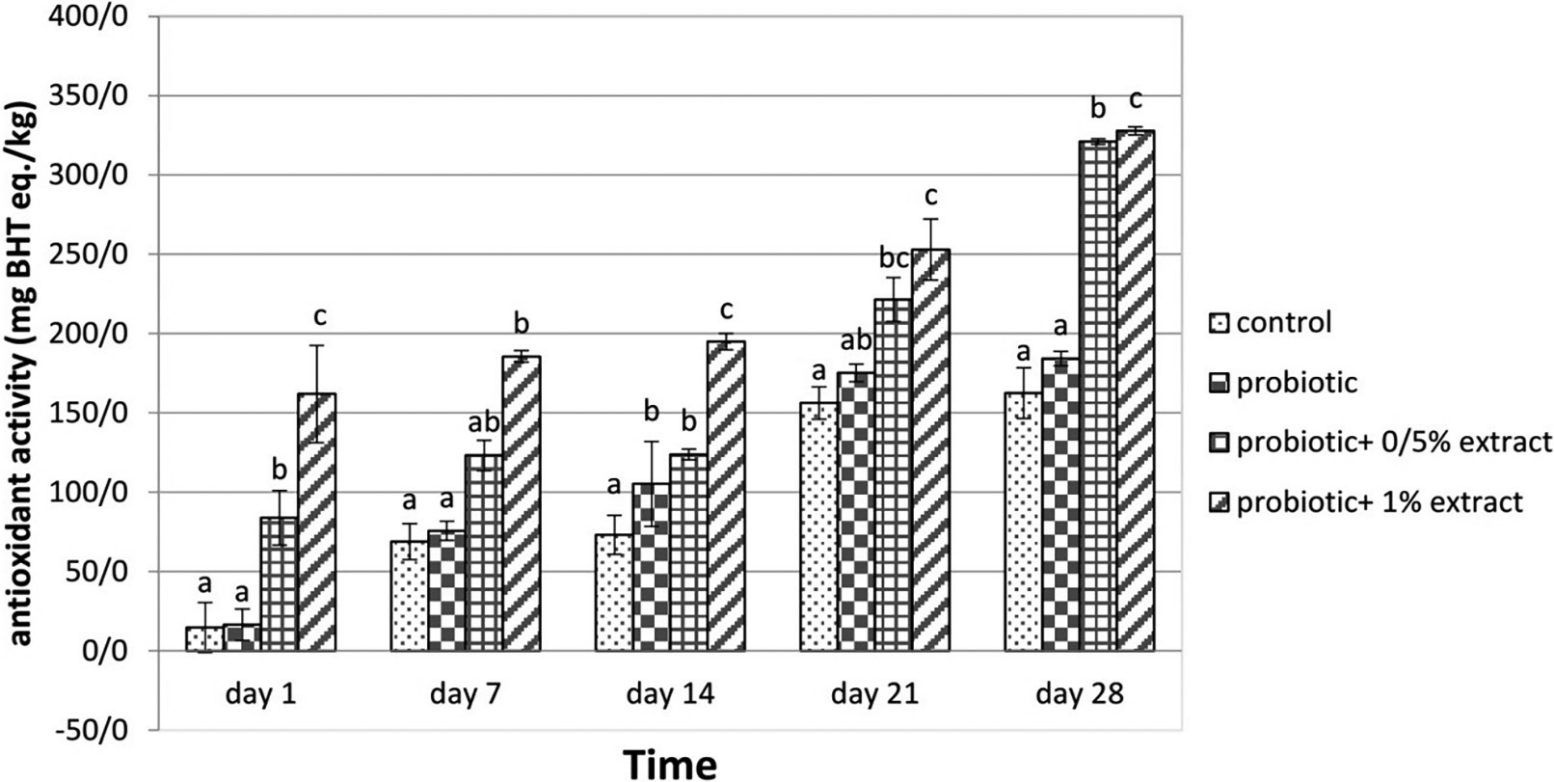
The effect of aqueous extract of *Arctium lappa* root (ALE) on antioxidant activity in studied groups during 28 days of storage at 4°C. Values followed by different lowercase letters on the same days are significantly different.

The antioxidant activity studied during the storage time is summarized in Figure 4. The samples of yogurt treated with probiotics containing 0.5% and 1% of ALE and the probiotic group showed significant differences in antioxidant activity. During the experiment time, the antioxidant activity of the probiotic yogurt group containing 0.5% and 1% of ALE increased from 83.8 and 155.1 mg BHT eq./kg on day 1 to 321 and 327.8 mg BHT eq./kg on day 28, respectively. Moreover, there was a strong positive correlation between antioxidant activity and ALE concentration (*r* = .822, *p* < .05). It should be noted that the antioxidant activity of ALE was 0.102 mg/mL.

#### 3.2.3 Titratable acidity

**FIGURE 5:**
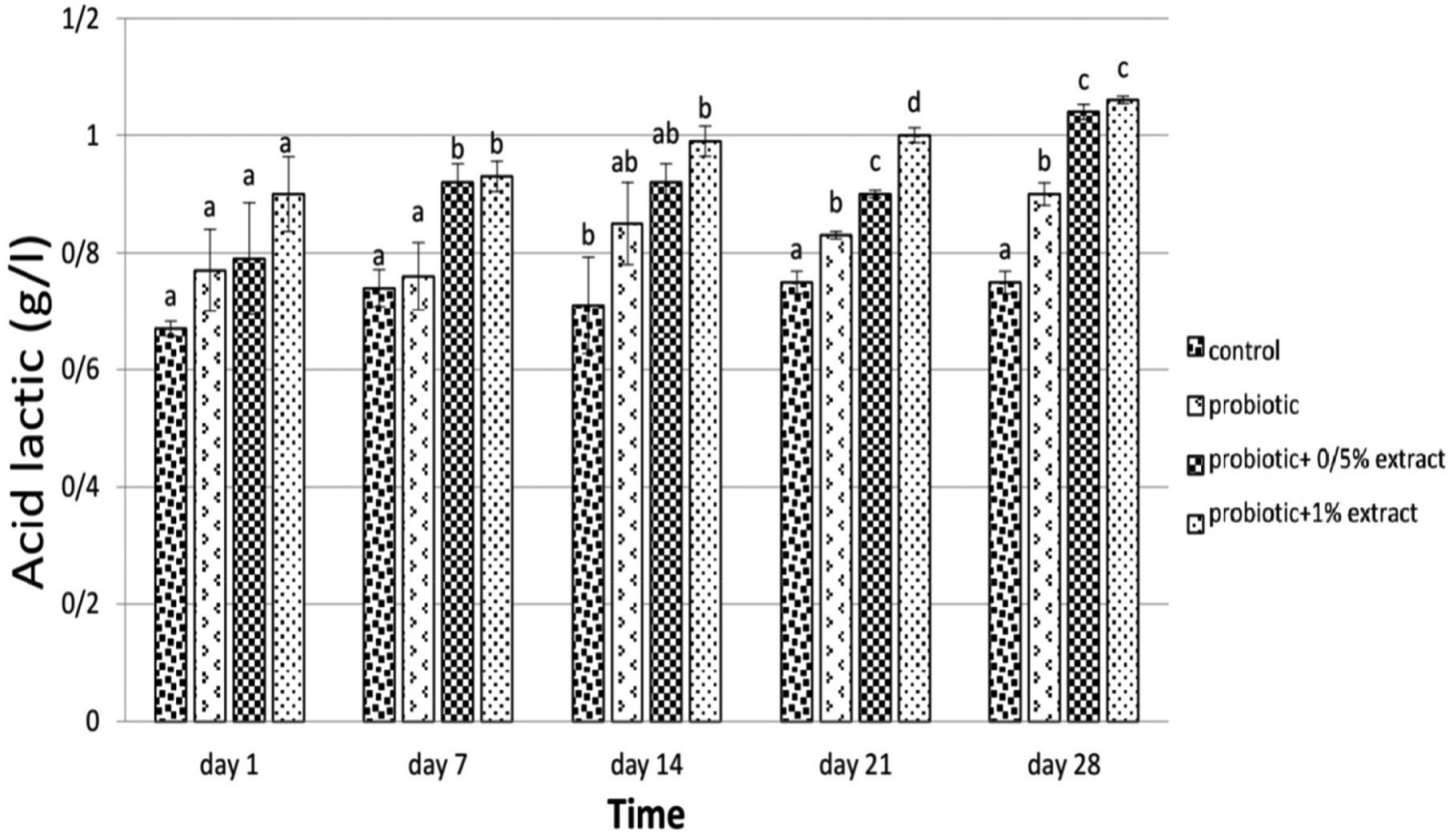
The effect of aqueous extract of *Arctium lappa* root (ALE) on titratable acidity (g lactic acid/L) in studied groups during 28 days of storage at 4°C. Values followed by different lowercase letters on the same days are significantly different.

First, it is worth mentioning that the titrable acidity (%) of ALE was 0.98. As shown in Figure 5, the results of the edge revealed that the bite in the group of yogurts containing probiotics on the 1st day was 0.77 and increased during the study to 0.90 on the 28th day, which shows a significant difference with the control group (*p* < .05). Adding 0.5% of ALE increased the acidity (from 0.79 on day 1 to 1.04 on the 28th day). With increasing the concentration of the extract to 1%, the edge reached 0.90 on day 1, and after 28 days, the acidity was calculated to be 1.06. Finally, no significant difference in sharpness was observed between the two groups with 0.5% and 1% of the extract (*p* > .05).

#### 3.2.4 pH

**FIGURE 6:**
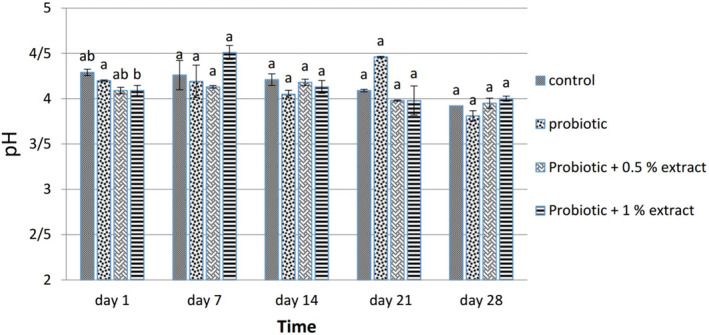
The effect of aqueous extract of *Arctium lappa* root (ALE) on pH in studied groups during 28 days of storage at 4°C. Values followed by different lowercase letters on the same days are significantly different.

Figure 6 depicts the result of the pH of different groups. During the study period, the pH of all groups decreased significantly (*p* < .05), but no significant difference was observed in comparing the pH results of different groups (*p* > .05). It should be noted that the pH of ALE was 5.83.

#### 3.2.5 Syneresis

**FIGURE 7:**
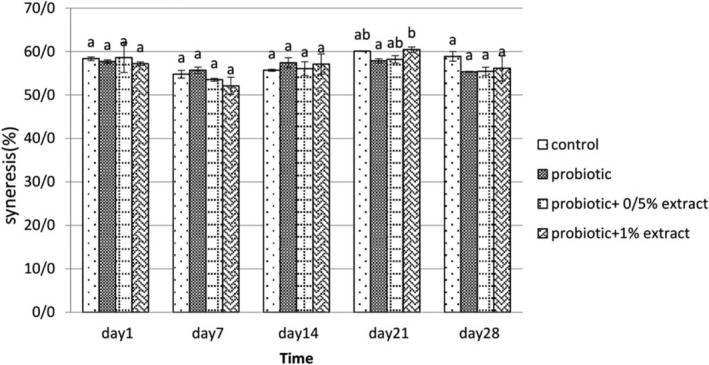
The effect of aqueous extract of *Arctium lappa* root (ALE) on syneresis (% w/w) in studied groups during 28 days of storage at 4°C. Values followed by different lowercase letters on the same days are significantly different.

The syneresis results of the studied groups during storage are summarized in Figure 7. Syneresis of control, probiotic yogurt, and 0.5% ALE‐containing yogurts have been reached from 58.4% to 58.9%, 57.46% to 55.33%, and 58.6% to 55.43% after 28 days of storage, respectively. With increasing the concentration of the extract to 1% compared to 0.5%, no significant change was observed in syneresis (*p* > .05). In general, a decrease in synergism was observed in all groups compared to the control group after 28 days.

#### 3.2.6 Sensory properties

**FIGURE 8:**
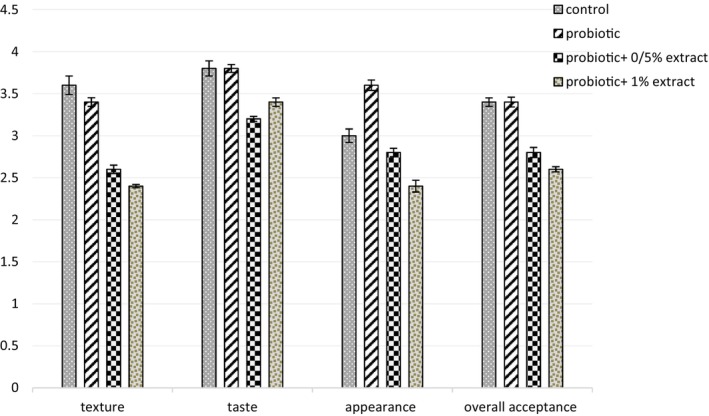
The effect of aqueous extract of *Arctium lappa* root (ALE) on sensory assessment in studied groups during 28 days of storage at 4°C. Values followed by different lowercase letters on the same days are significantly different.

The data in Figure 8 depict the sensory evaluation results of the control and different treatment groups of yogurt. No significant difference was observed between the apparent rank of the power and probiotic yogurt (*p* > .05). While adding 0.5% of the extract to the yogurt changed the color from white to creamy, it reduced its apparent acceptance, and with increasing concentration to 1%, this color change increased, so the evident rank in this group decreased to 2.4. The results indicated that the control and probiotic yogurt groups received the highest scores in texture. Adding 0.5% and 1% ALE caused a relative decrease in yogurt viscosity and softer yogurt texture. There was no significant difference between the control group and probiotic yogurt regarding taste and overall acceptability (*p* > .05). Adding ALE to yogurt changed the taste of yogurt to sweet; in the general acceptability of the probiotic yogurt, 0.5% received a score of 2.8, and in probiotic yogurt, 1% of ALE received a score of 3.

## 4 DISCUSSION

### 4.1 Survival of probiotics

The survival of probiotic bacteria La‐5 and Bb‐12 in synbiotic yogurt containing ALE was evaluated, and the results showed that the ALE had no significant reduction effect on probiotic bacteria (*p* > .05). Consideration of the survival of La‐5 during storage revealed that from the beginning of the study to the 21st day, there was no significant difference in the survival of this bacterium in the presence and absence of ALE, but on the last day, the difference was significant and the amount of bacteria decreased in the treatment without ALE (*p* < .05), but in the study of survival, Bb‐12 was found to be the highest amount counted on the 7th day of the study, but no significant difference was observed between the probiotic treatment without ALE and the treatment with the extract (*p* > .05), and a decrease in the survival of bacteria was reported in the following days. The prebiotic performance of ALE has been investigated in vitro and in vivo, but very few studies have been performed on food. Park et al. (2007) identified active compounds such as tannin, actinin, caffeic acid, chlorogenic acid, inulin, trachelogenin, sitosterol, beta‐D‐glucopyranoside, lapaul, and arctigenin in ALE (Gomes & Malcata, 1999). Listed oligosaccharides in *Arctium lappa* as one of the factors influencing the growth of various species of *Bifidobacterium* and classified it as a bifidogenic compound. In addition to inulin, which is present in significant amounts in the roots of *Arctium lappa*, other compounds such as various vitamins and essential amino acids have been identified in this plant that can play a prebiotic role. For example, Kolida and Gibson (2007) and Roberfroid (2005) stated that foods containing probiotics and inulin could be considered functional foods that, in addition to having high nutritional value, also have an anticancer effect (especially colon cancer). Studies conducted by Knöbel et al. (1997) on the prebiotic effect of ALE in broilers showed that the prebiotic effect of inulin in the extract of this plant increases the growth of Bifidobacteria in the intestines of broilers. Bifidobacteria use inulin and other fructooligosaccharides in this extract as raw materials in fermentation and inhibit the growth of pathogenic bacteria, which ultimately increases production efficiency. It should be noted that the beneficial effects of bifidobacteria are due to the production of immune‐stimulating compounds and the synthesis of B vitamins. Lee et al. (2006) investigated the prebiotic effects of inulin extracted from ALE under in vitro and in vivo conditions. In the presence of inulin, the growth of Bifidobacteria and Lactobacillus increased sharply. Their results indicated the health effects of inulin from ALE (Rao, 1999) and evaluated the prebiotic activity of 1% inulin based on the growth rate of probiotic species compared to 1% glucose under the same conditions after 24 h. They announced that the highest growth rate of *Lactobacillus paracasei* was on inulin, which was significantly higher than the growth on glucose, and the lowest growth rate was related to the development of Bifidobacterium on galactooligosaccharide. In this context, inulin was found to be a suitable substrate for the growth of Bifidobacteria compared to glucose. Also, according to the observations of Akın et al. (2007) in a study on inulin‐enriched probiotic ice cream, the bacterial count in the control samples was less than the samples enriched with inulin, which indicates that the presence of inulin stimulated the growth of *Lactobacillus acidophilus* and *Bifidobacterium bifidum*.

### 4.2 Total phenolic content and antioxidant properties

In the present study, adding an aqueous extract of ALE to yogurt increased the phenolic content, so that the phenolic content was significantly higher in the treatment containing 1% ALE during the study and especially on day 14th compared to other treatments and after that the treatment containing 0.5% ALE (*p* < .05). The reason for this can be the presence of chlorogenic acid and its derivatives, lignans, especially actinin, and various flavonoids.

The results of a study by Duh (1998) showed that caffeic acid and flavonoids such as quercetin, which are found in the ALE, have higher antioxidant activity than BHA and BHT.

They also reported that in extracting from different parts of this plant, the phenolic content directly affects the antioxidant function, and the phenolic compounds present in ALE cause antioxidant activity.

Phenolic compounds can act as antioxidants by transferring hydrogen or electrons and terminating a chain reaction or chelating metal ions (Rice‐Evans et al., 1997). The present study's results showed that adding ALE to yogurt increases antioxidant activity. Additionally, during the incubation period, the treatment containing 1% and then the treatment containing half percent ALE showed a continuous and significant increase in antioxidant activity compared to other treatments. This increase is due to the high antioxidant potential of caffeic acid and quercetin. Milk also has some antioxidant activity due to its constituents as bioactive peptides, which are increased in fermented milks. On the other hand, bioactive peptides are released as a result of the proteolytic activity of lactic acid‐producing bacteria in fermented milk products and cause antioxidant activity (Farvin et al., 2010).

Various studies have shown that natural antioxidants may prevent oxidation and benefit human health—for example, the antiaging, anticancer, and antimutagenic effects. In the present study, it was found that the antioxidant activity of yogurt‐containing ALE increased during storage, which could be due to the decomposition of compounds of ALE, the production of compounds with higher antioxidant activity, and the reaction of proteolysis compounds in milk with phenolic composition of the extract, which increases the antioxidant activity. Kudoh et al. (2001) demonstrated in a study that fermented milk with *Lactobacillus bulgaricus* increased the antioxidant properties of yogurt due to the production of κ‐Casein peptides that have DPPH radical inhibitory activity, which is consistent with the results of the present study.

### 4.3 Acidity and pH

During the storage of yogurt‐containing ALE, the amount of acidity increased. These results are consistent with a 2016 study (Jirsaraei et al., 2016). They investigated the effect of inulin addition on physicochemical parameters such as acidity and pH in probiotic feta cheese for 60 days. They reported that the trend of pH changes for all cheese samples during the storage was downward. Conversely, the acidity of probiotic cheese samples gradually increased significantly during storage, which is in line with the results of the present study. Pourahmad & Shaghaghi (2016) recorded inulin as a stimulant of probiotic metabolic activity and consequently increased acidity. This study's results were consistent with studies by Donkor et al. (2007) and Gouin (2004).

### 4.4 Syneresis

As observed, adding ALE to yogurt reduced the undesirable features of the syneresis of the samples. No significant change in syneresis was marked by increasing its concentration to 1% in yogurt samples. However, a downward trend was observed compared to the control group due to the formation of a denser gel network compared to the control sample due to the adsorption of hydrocolloid water in the samples (Staffolo et al., 2004). Also, the lowest amount of syneresis during storage was on the 7th day and related to the treatment of 1% ALE.

Brennan and Tudorica (2008) also stated in their study that the addition of *Plantago ovata* seed mucilage has reduced the syneresis of low‐fat yogurt. Sharabiani et al. (2010) also reported a decrease in syneresis by increasing the gum concentration in yogurt. They added about 1.3% of inulin to yogurt, and it was found that yogurts showed a slight change in syneresis even after 21 days of storage at 4°C. However, there was a more significant increase in the control sample. This is probably due to the presence of a gel network in the samples containing inulin, which traps water and casein micelles, and the absence of such networks was mentioned in the control sample.

### 4.5 Sensory properties

As it was determined in the sensory results of this study, probiotic yogurt samples in which there was 0.5% and 1% ALE obtained lower acceptance in texture and appearance characteristics than the control group. However, the taste of yogurt samples containing ALE due to its sweetness (due to high inulin in ALE) obtained a higher score than the appearance and texture of the control group. It led to improved oral sensation, which can be caused by creaminess. Creamy is due to the formation of gel crystals. The low score related to the appearance is the observation of brownish cream color in the samples containing ALE, which was because the root peeling of this plant was not performed. Staffolo et al. (2004) evaluated the effect of some dietary fiber on the sensory properties of yogurt and found that samples containing inulin had the highest taste score, which is in line with the results of the present study (Bosnea et al., 2017). Examined the effect of inulin on the sensory properties of probiotic yogurt and concluded that inulin in probiotic yogurts did not show a significant difference in texture, taste, and color compared to the control group.

### 4.6 Practical discussion

The use of prebiotics in dairy products containing probiotics, in addition to increasing the viability of probiotic bacteria, causes health‐enhancing effects in the consumer, and as a rich source of fibers, it can have beneficial effects such as solving digestive problems and reducing blood cholesterol and blood sugar (Rahmani et al., 2021). The results of the present study also showed that the extract of the papaya plant could act as a stimulant for the growth of probiotics, and due to the presence of fibers such as inulin, the beneficial effects of fiber consumption follow.

## 5 CONCLUSION

As mentioned, two concentrations of aqueous extract of *Arctium lappa* root (0.5% and 1%) were used, and sensorial and physicochemical properties of yogurt were evaluated during 4 weeks of storage. The results showed that the survival of *Lactobacillus acidophilus* La‐5 and *Bifidobacterium bifidum* Bb‐12 has been improved during the warehouse. Moreover, adding an aqueous extract of *Arctium lappa* root to yogurt enhanced antioxidant activity and phenolic content to 1299.8 mg gallic acid /kg and 392.8 mg BHT eq./kg after storage, respectively. In general, in synbiotic yogurt, a decrease in syneresis, undesirable changes in taste, texture, and appearance, and reduced overall acceptance were observed. According to the results of the present study, using an aqueous extract of *Arctium lappa* root can improve nutritional properties and probiotic protection in yogurt during long time storage; thus, it is a good choice for application in the dairy industry.

## AUTHOR CONTRIBUTIONS

**Elmira Vanaki:** Investigation (equal). **Abolfazl Kamkar:** Project administration (equal). **Negin Noori:** Writing – original draft (equal). **Asghar Azizian:** Software (equal). **fatemeh Mohammadkhan:** Writing – review and editing (equal).

## CONFLICT OF INTEREST STATEMENT

There are no conflicts of interest to declare.

## ETHICS STATEMENT

This study does not involve any human or animal testing.

## Data Availability

All data generated and analyzed during this study are available from the corresponding author at a reasonable request.
